# Clinical and genetic characteristics of 29 Chinese patients with X-linked hypophosphatemia

**DOI:** 10.3389/fendo.2022.956646

**Published:** 2022-08-19

**Authors:** Tian Xu, Xiaohui Tao, Zhenlin Zhang, Hua Yue

**Affiliations:** Shanghai Clinical Research Center of Bone Diseases, Department of Osteoporosis and Bone Diseases, Shanghai Jiao Tong University Affiliated Sixth People’s Hospital, Shanghai, China

**Keywords:** X-linked dominant hypophosphatemia, *PHEX*, fibroblast growth factor 23, clinical features, gene mutation

## Abstract

**Objective:**

The aim of this study was to fully describe the clinical and genetic characteristics, including clinical manifestations, intact fibroblast growth factor 23 (iFGF23) levels, and presence of *PHEX* gene mutations, of 22 and 7 patients with familial and sporadic X-linked dominant hypophosphatemia (XLH), respectively.

**Methods:**

Demographic data, clinical features, biochemical indicators, and imaging data of 29 patients were collected. All 22 exons and exon–intron boundaries of the *PHEX* gene were amplified by polymerase chain reaction (PCR) and directly sequenced. The serum level of iFGF23 was measured in 15 of the patients.

**Results:**

Twenty-nine patients (male/female: 13:16, juvenile/adult: 15:14) with XLH were included. The main symptoms were bowed lower extremities (89.7%), abnormal gait (89.7%), and short stature/growth retardation (78.6%). Hypophosphatemia with a high alkaline phosphatase level was the main biochemical feature and the median value of serum iFGF23 was 55.7 pg/ml (reference range: 16.1–42.2 pg/ml). Eight novel mutations in the *PHEX* gene were identified by Sanger sequencing, including two missense mutations (p. Gln682Leu and p. Phe312Ser), two deletions (c.350_356del and c.755_761del), one insertion (c.1985_1986insTGAC), and three splice mutations (c.1700+5G>C, c.1966-1G>T, and c.350-14_350-1del). Additionally, the recurrence rate after the first orthopedic surgery was 77.8% (7/9), and five of them had their first surgery before puberty.

**Conclusion:**

Our study expanded the clinical phenotypes and gene mutation spectrum of XLH and provided a reference for the optimal timing of orthopedic surgeries.

## Introduction

X-linked hypophosphatemia (XLH, OMIM 307800) is characterized by disordered phosphorus metabolism and abnormal bone mineralization, with an estimated prevalence of 1 in 20,000 and an approximate incidence of 3.9–5 per 100,000 live births (1). XLH is a disorder caused by *phosphate regulating endopeptidase homolog X-linked* (*PHEX*) gene mutations transmitted in an X-linked dominant manner, and is found in 80% of patients with inherited rickets/osteomalacia (2). The *PHEX* gene encodes a transmembrane endopeptidase that belongs to the type II integral membrane zinc-dependent endopeptidase family and is predominantly expressed in bone and teeth with cellular localization in osteoblasts, osteocytes, odontoblasts, and ameloblasts (3, 4).

Loss-of-function mutation of the *PHEX* gene results in downregulation of the PHEX protein and increased circulating levels of fibroblast growth factor 23 (FGF23). PHEX protein is an enzyme that degrades local small integrin-binding ligand, N-linked glycoproteins (SIBLING proteins), especially osteopontin. Deposition of osteopontin in bone on account of decreased expression of PHEX enzyme affects local inhibition of mineralization (5). FGF23, a critical phosphatonin that is almost exclusively produced by osteocytes and osteoblasts, regulates phosphate and vitamin D homeostasis. Excess FGF23 inhibits renal phosphate reabsorption by reducing the expression of the renal tubular sodium phosphate cotransporters. Meanwhile, overexpression of FGF23 lowers 1,25(OH)_2_D_3_ by regulating the expression of *CYP27B1* and *CYP24A1*, reducing the synthesis and enhancing the catabolism of 1,25(OH)_2_D_3_ (5–7). Although FGF23 is not the direct substrate of the PHEX enzyme, a recent study indicated that PHEX is a direct transcriptional inhibitor of FGF23, which plays an important role by affecting the expression of FGF23 rather than the degradation of FGF23 (7).

The clinical manifestations of XLH vary between children and adults. Gait abnormality, lower-extremity deformity, growth retardation, and rickets are common in pediatric patients. In contrast, disproportionately short stature, osteomalacia, bone pain, osteoarthritis, pseudofractures, pathological fractures, enthesopathy, and dental problems are the main complaints in adult patients. Lower-extremity deformity was reported as the most frequent clinical manifestation in patients of all ages, which delays the growth of the long bones and then worsens the reduction in height (8). As shown in the literature, 24%–65% of patients with XLH required surgical intervention for lower-extremity deformities (9, 10). Some of them underwent orthopedic surgeries to obtain a neutral axis of the lower limbs. However, the best timing and approach for corrective osteotomies in XLH patients continues to constitute a matter of debate (1, 11).

Recently, burosumab, a recombinant human monoclonal IgG1 antibody against FGF23, became available in China. Burosumab can bind and inhibit excess FGF23 and thereby corrects hypophosphatemia and ameliorates clinical symptoms in XLH patients. Published data from phase 2 and 3 clinical trials showed that burosumab improved renal tubular phosphate reabsorption, serum phosphorus levels, and linear growth, and reduced pain and the severity of rickets in children (12, 13). In addition to restoring normal serum phosphate concentrations, burosumab significantly alleviated osteoarticular stiffness, helped to heal fractures, increased levels of bone turnover markers (BTMs), and improved osteomalacia-related histomorphometric measures in adult patients (14, 15). Compared with those receiving conventional treatment, pediatric patients treated with burosumab showed greater improvements in rickets severity, growth, and biochemistry (16).

Apart from acting on bone and kidney, FGF23 might have a potential long-term influence on the cardiovascular and metabolic system as some studies reported (17, 18). Although FGF23 measurement is not recommended during the follow-up, it is valuable to investigate the relationships between the levels of FGF23 and genotypes, long-term complications, and the prognosis (1). To date, more than 800 different *PHEX* mutations have been identified (HGMD, http://www.hgmd.cf.ac.uk/ac/index.php and https://www.rarediseasegenes.com) (19). In our study, we fully described the clinical features, *PHEX* gene mutation sites, and iFGF23 levels of 29 patients with XLH as well as recommend optimal operation timing.

## Materials and methods

### Subjects

Our study was approved by the Ethics Committee of Shanghai Jiao Tong University Affiliated Sixth People’s Hospital and informed written consent was obtained from all patients or their guardians (for participants who were under 18 years old). We retrospectively analyzed the clinical characteristics and *PHEX* gene mutation profiles of 29 patients with XLH from January 2020 to February 2022. Altogether, there were 29 individuals from 20 families, including 22 familial and 7 sporadic cases. The exclusion criteria were as follows: hypophosphatemia with other causes, such as autosomal dominant or recessive hypophosphatemic rickets, defects in intrinsic renal phosphate transport, and secondary hypophosphatemia, such as tumor-induced osteomalacia, and drug-induced and nutrition-induced hypophosphatemia. Only one adult patient received burosumab subcutaneous injection with 1.0 mg/kg per month, whose primary manifestations were pain of lower extremity and elevated level of ALP before treatment. The remaining patients received conventional treatment with oral phosphate 20–40 mg/kg and calcitriol 0.25–0.5 μg per day since they came to our clinic. Both calcitriol and alfacalcidol are analogs of highly active vitamin D, which can be used to treat XLH (11).

### Clinical features

The height data were converted into a standard deviation score (SDS) according to standardized growth charts for children and adolescents in China (20). For children, bilateral posteroanterior wrist and knee radiographs were available, and the rickets severity score (RSS) was evaluated according to the method of Thacher (21). The RSS is scaled incrementally from 0 to 10, corresponding to normal to severe rickets. For adults, necessary radiographs were taken if bone or joint pain was present.

### Biochemical measurements and serum intact FGF23 measurement

Biochemical indicators included serum phosphate, total calcium, total alkaline phosphatase (ALP), intact parathyroid hormone (iPTH), 25-hydroxyvitamin D (25OHD), β-isomerized C-terminal telopeptide of type 1 collagen (β-CTX), and serum osteocalcin (OC). Biochemical indexes and BTMs were measured by a Hitachi 7600-020 automatic biochemistry analyzer and an automated Roche electrochemiluminescence system, respectively. Blood samples of 29 patients were drawn in the morning after fasting overnight.

Serum iFGF23 levels were measured by using a two-site ELISA kit (KAINOS Laboratories, Inc., Tokyo, Japan) with a detectable concentration range from 3 to 800 pg/ml. The reference range was 16.1–42.2 pg/ml (22).

### Sanger sequencing for *PHEX* gene mutation detection and pathogenicity prediction

Genomic DNA was extracted by a conventional method using a DNA extraction kit (Lifefeng Biotech, Shanghai). The DNA sequence of the *PHEX* gene was obtained from an online database (GenBank accession No. NC_000012). All 22 exons with their adjacent intronic sequences of the *PHEX* gene were amplified by PCR with 21 pairs of sequencing primers designed using Primer 3 software. The primers were presented in **Table 1**. Direct DNA sequencing was performed using a BigDye Terminator Cycle Sequencing Ready Reaction Kit, version 3.1 (Applied Biosystems, Foster, CA, United States), and then analyzed with an automated ABI 3730 sequencer (Foster, CA, United States). Single-nucleotide polymorphisms (SNPs) were identified using Polyphred2, and novel mutations were identified using HGMD (23). Polyphen-2 (http://genetics.bwh.Havard.edu/pph2/), PROVEAN (http://provean.jcvi.org/index.php), and Mutation Taster (http://www.mutationtaster.org/) were used to predict the pathogenicity of missense mutations in the *PHEX* gene. For Polyphen-2 analysis, the following three empirically derived outcomes were used: most likely damaging, possibly damaging, and benign. For PROVEAN, scores ≤−2.5 are predicted to be deleterious. For Mutation Taster, the possibility of a mutation being a disease-causing mutation is scaled from 0 (low) to 1 (high).

**Table 1:** Primer sequences for PCR amplification of the *PHEX* gene.

Exons	Forward primer (5’→3’)	Forward primer (5’→3’)
1	AGGGACTTTGCTGAGGGAGAG	CCACTCGAAGCCACTTACACC
2	TGGGTTTTGGAATACCGTGTC	AAGAGAGGCCATTCAGCCTTC
3	CAAGGCTTGGAAACTGGTTGA	TTATGTTGAGATCTGGGAGTCCA
4	GGCACCATATGTGGGTGGATA	GTTTGCCCTGCTGACTTTGTC
5	CACATTGAAGCGTGGATCGTA	CGGGAGAAGGGAATATTCTGG
6	GCTCTGCCCAATCATGTTACC	GCAGCCTGGTAAGGCACATAG
7	GGGTGCCTGGTATTGCATAAT	CCAATGGGCAATGACACAAA
8	ACCACACCAAAGCCTTGAAAA	GAGCCAATGCCAACAATTACC
9	GGATGGCAATGATCAGGAGTT	GACAGTGCTTTTGGCCAGTTC
10	ATGTTCACTCTGAGGGCTGGA	GGCTACAAACTCCCCCTGTCT
11	CAGCCATGGGTTTTATCCAAA	CCCACTCCCCTGGAAAACTAC
12	AGTGTTGCCAGAGCATGGAGT	AGGAAAGGCCGAATTACAAGG
13	TCGATTCAGTCACCTTCTCCA	GAAAGGCACAAGGCCAGTAAA
14	TGACTGATGCAGCTTCTCTGC	ATGCTAGAAATGGGGGACCTG
15	GCAGGGACAGCCCTTTAGATT	GCCACTTTTGGGGGAAATAAG
16	GTGCAAAATGGTTTCCCTGAA	GTCCAGCCATACACCCTGGTA
17	AAGCAGTTTATCTTGGCTTTCCA	CAAGCCATCACAGCAAGACAC
18	CTGCTTTTTGAAGGCTTGTCG	ATGCCTGGTTAAGGGATGACC
19	TTGATGCCTCTTGCTGAATGA	AAATGAACCTAGCCCCAAGGA
20	TGGTAAGCAACAGGACATGGA	AGGGCTGCTAACCCATTTGAT
21	TTCCTGGGCACATATACGATTC	TTTTGGCTGCAAAATGGAAAT
22	CAGAACCTGTTGATGTGCAAGA	GCCAACACCCTAAAATGGACA

PCR, polymerase chain reaction; PHEX, phosphate-regulating gene with homology to endopeptidase on the X chromosome.

### Statistical analyses

All data were analyzed by using IBM SPSS Statistics (version 23.0; SPSS Inc., Chicago, IL, United States). The normality of the distribution of continuous variables was checked by the Kolmogorov–Smirnov test. Normally distributed, nonnormally distributed, and categorical variables are presented as the mean ± SD, medians (25th and 75th percentiles), and frequencies or percentages, respectively. Intergroup differences for such variables were assessed with independent-sample *t* tests, the Mann–Whitney *U* test, and Fisher’s exact test, respectively. Correlations between continuous variables were evaluated with the Spearman rank correlation coefficient. *p* < 0.05 was considered statistically significant.

## Results

### Clinical features of the subjects

**Figure 1:**
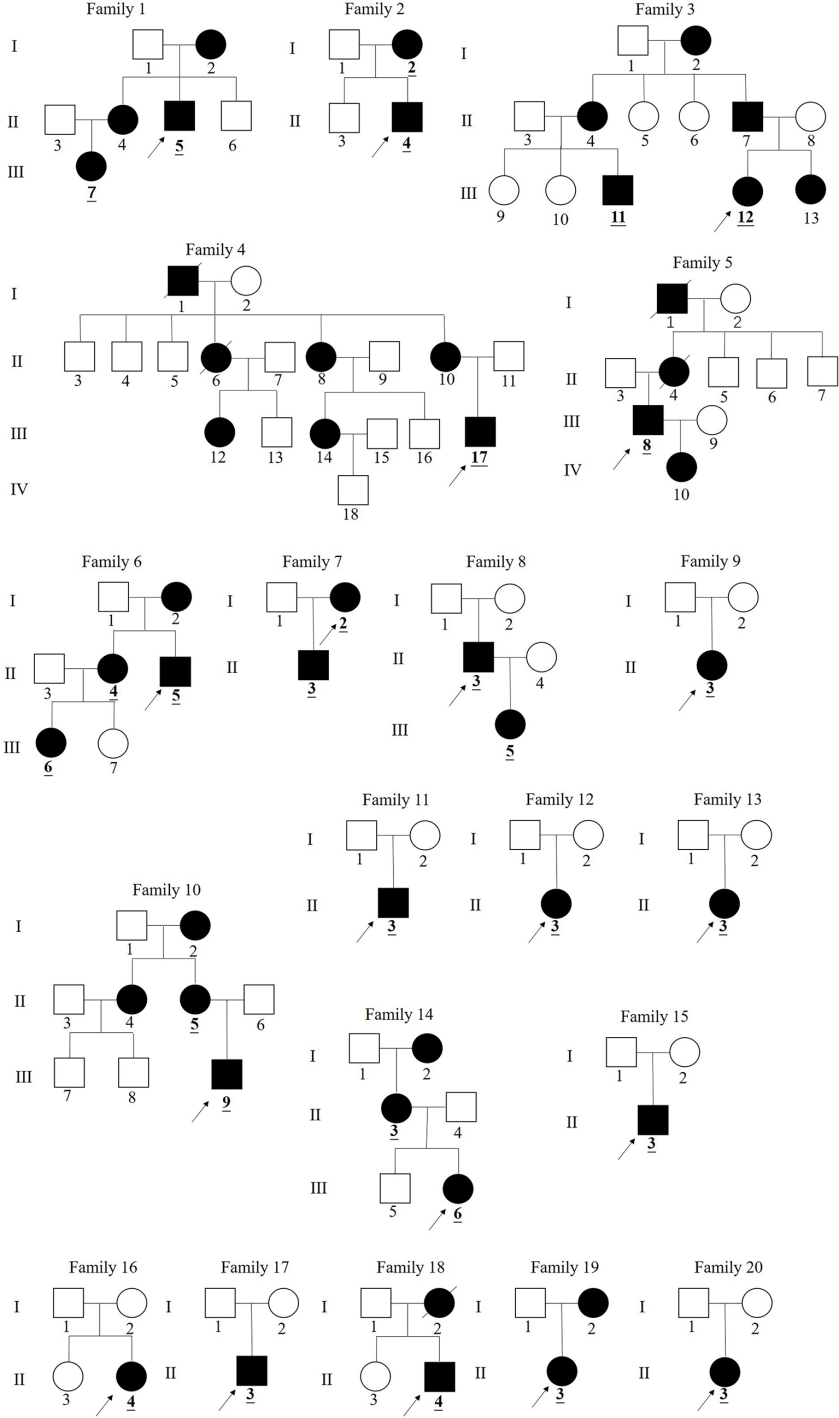
Pedigree of families 1–20 in China with X-linked hypophosphatemia. (The black symbols represent the affected individuals and the open symbols represent the unaffected individuals. The circles and squares indicate female and male, respectively. The arrows identify the proband in the families.The numbers underlined and bolded in pedigrees identify 29 patients in this study).

Pedigrees of the XLH patients are shown in **Figure 1**. Twenty-nine patients (female:male, 16:13), specifically 7 sporadic and 22 familial cases, were recruited in our study, including 15 juveniles and 14 adults, with an average age of 18.5 years (18.5 ± 14.5 years) and a median onset age of 24 months (12–36 months). The average height SDS in adult and juvenile patients was −3.9 ± 2.8 and −1.3 ± 1.2, respectively. Short stature was more significant in adults than in juveniles (*p* < 0.01). Clinical complaints at the initial visit mainly included abnormal gait (89.6%), deformities of weight-bearing limbs (89.6%), short stature/growth retardation (78.6%), dental problems (53.6%), bone pain (37.9%), and a history of pseudofractures or pathological fractures (17.2%). The average RSS was 4.3 points (4.3 ± 1.3) in nine juveniles with available radiographs, indicating the impairment of skeletal development. The detailed demographic data and clinical features are provided in **Table 2** and **Supplementary Table 1**.

**Table 2:** Clinical characteristics and complications of 15 pediatric patients and 14 adult patients with XLH.

Clinical characteristics	Pediatric patients	Adult patients	*p*-value
Sex (female: male), *n*	8:7	8:6	–
Age, years	6.5 ± 4.6	31.4 ± 9.2	–
Age of onset, months	18 (12, 18)	24 (12, 39)	0.860
Height (SDS)	−1.3 ± 1.2	−3.9 ± 2.8	**0.027**
BMI, kg/m^2^	17.4 (16.3, 19.0)	25.7 ± 4.9	**0.001**
iFGF23, pg/ml	95.7 ± 90.8	48.0 (31.5, 86.8)	0.864
Bowed lower extremities	12/15 (80.0%)	14/14	0.238
Abnormal gait	12/15 (80.0%)	14/14	0.238
Short stature, growth retardation	8/14 (57.1%)	14/14	**0.016**
Dental problems	2/15 (13.5%)	13/14 (92.9%)	**0.001**
Bone pain	1/15 (0.7%)	10/14 (71.4%)	**0.001**
Pseudofracture/Fracture	1/15 (0.7%)	4/14 (28.6%)	0.169
Orthopedic surgery	3/15 (20%)	9/14 (64.3%)	**0.025**
Treated : Untreated, *n*	10:5	8:6	–
Nephrocalcinosis, %	0 (0/6)	25% (2/8)	0.473
Secondary hyperparathyroidism, %	50% (7/14)	58.3% (7/12)	0.713
Ectopic ossification, *n*	–	4	–

Values were shown as n, means ± SD or median (25th, 75th). The meaning of the bold values represented 29 patients.

### Biochemistry and serum iFGF23 levels of XLH

The biochemical indicators of the 29 patients are summarized in **Table 3**. For all age groups, the mean serum phosphorus and creatine levels were below the lower limit of the normal range, while the level of ALP was above the upper limit, and BTMs were high. Nine patients had vitamin D deficiency (25OHD < 20 ng/ml) with slightly elevated iPTH levels. The average level of vitamin D in adult patients was significantly lower than that in juveniles (*p* < 0.01). The levels of iFGF23 were detected in 15 patients, with a median value of 55.7 pg/ml (range: 24.0–146.0 pg/ml). A total of 53.3% of patients had a high level of iFGF23 above the upper limit of the reference range (42.2 pg/ml). However, neither significant difference between adult and juvenile patients nor correlations between the levels of iFGF23 and height SDS, RSS, BMI, or age of onset were detected (*p* > 0.05).

**Table 3:** Biochemical features of patients with XLH.

Biochemical parameters	Age groups	Sample number (*N*)	
Serum phosphorus, mmol/L	<1 years	2	1.00, 1.00
	1–3 years	3	0.84, 0.75, 0.85
	4–11 years	8	0.94 ± 0.13
	12–15 years	1	0.67
	>15 years	13	0.59 ± 0.11
Serum calcium, mmol/L		27	2.30 (2.28, 2.42)
ALP, U/L	1–15 years	14	594.1 ± 161.0
	16–18 years	1	302.0
	>18 years	11	124.0 (75, 172)
PTH, pg/ml		25	71.72 (48.67, 100.00)
25OHD, ng/ml		26	27.59 ± 16.48
iFGF23, pg/ml		15	55.7 (24.0, 146.1)
β-CTX, ng/L		23	1,569.07 ± 982.17
OC, ng/ml		22	60.75 (24.06, 107.45)
Serum creatine, μmol/L		26	37.98 ± 10.80

Values were shown as n, means ± SD or median (25th, 75th). ALP, total alkaline phosphatase; PTH, parathyroid hormone; 25OHD, 25-hydroxy-vitamin D; β-CTX, β-isomerized C-terminal telopeptide of type 1 collagen; OC, serum osteocalcin in the form of N-terminal mid-molecule fragments. Normal reference range: phosphorus [varies by age]: 1–3 years: 1.25–2.10 mmol/L, 4–11 years: 1.20–1.80 mmol/L, 12–15 years: 0.95–1.75 mmol/L, >15 years: 0.80–1.60 mmol/L, calcium: 2.08–2.60 mmol/L, ALP: 1–15 years: 42–390 U/L, 16–18 years: 52–171 U/L; >18 years: 15–112 U/L (22), PTH: 15–65 pg/ml, 25OHD: >30 ng/ml, iFGF23: 16.1–42.2 pg/ml (22), β-CTX: 278–540 ng/L, OC: 13.07–27.68 ng/ml (24), creatinine: 53.0–115.0 μmol/L.

### Orthopedic surgeries and postoperative recurrence rates

Twelve patients with XLH underwent at least one orthopedic surgery, including three pediatric and nine adult patients. The three children underwent the surgeries within 6 months, including two children receiving transient hemiepiphysiodesis and the remaining one having the osteotomy. The detailed information of the nine adults who underwent orthopedic surgeries is summarized in **Table 4**. The mean age of the first operation was 11.2 years (11.2 ± 4.8 years) and the average height SDS was −4.1 ± 1.6. Most of the patients (6/9, 66.7%) underwent osteotomies. Nine adults were divided into three groups (prepuberty, adolescence, and post-puberty) according to the age of the first operation, with five, three, and one patient in each group (25). Except for one patient who underwent surgery post-puberty and another patient who had surgery in adolescence, seven out of nine patients who received corrective orthopedic surgeries had recurrent lower limb deformities. Two patients who underwent the first surgery before puberty recurred but achieved a satisfactory outcome after the surgery performed post-puberty. As to the operative approaches, one patient had unilateral operation due to varus in the left lower limb, and the remaining eight patients had bilateral corrective surgeries. Two patients who underwent surgeries at the age of 13 and 16 were administered oral phosphate and calcitriol before surgery. They have been satisfied with the effect of the corrective surgeries so far (**Table 4**).

**Table 4:** Orthopedic surgery of nine adults.

Family no.	Age	Age of operation	Lower-extremities deformity	Type of surgery	Postoperative satisfaction	Height (SD)
1/II-5/M	27	6/8/30	Valgus	Osteotomy	Y	−5.84
2/I-2/F	44	12	Valgus/varus^a^	NA	N	−5.66
6/II-5/M	24	10	Varus	Osteotomy	N	−6.73
6/II-4/F	30	6	Varus	Osteotomy	N	−3.09
7/I-2/F	34	22	Varus	Osteotomy	Y	−2.10
11/II-3/M	21	9/16^*^	Valgus	Osteotomy	Y	−3.52
12/II-3/F	27	10	Varus	NA	N	−2.99
14/III-6/F	24	13^*^	Varus	Osteotomy	Y	−3.09
20/II-3/F	23	13/23	Varus^b^	Hemiepiphysiodesis	N	−3.68

F, female; M, male; NA, not available; N, no; Y, yes. ^a^valgus in right lower limb and varus in left lower limb; ^b^varus in the left lower limb; *a history of medication before surgery.

### Manifestations and complications in 14 adults

**Figure 2:**
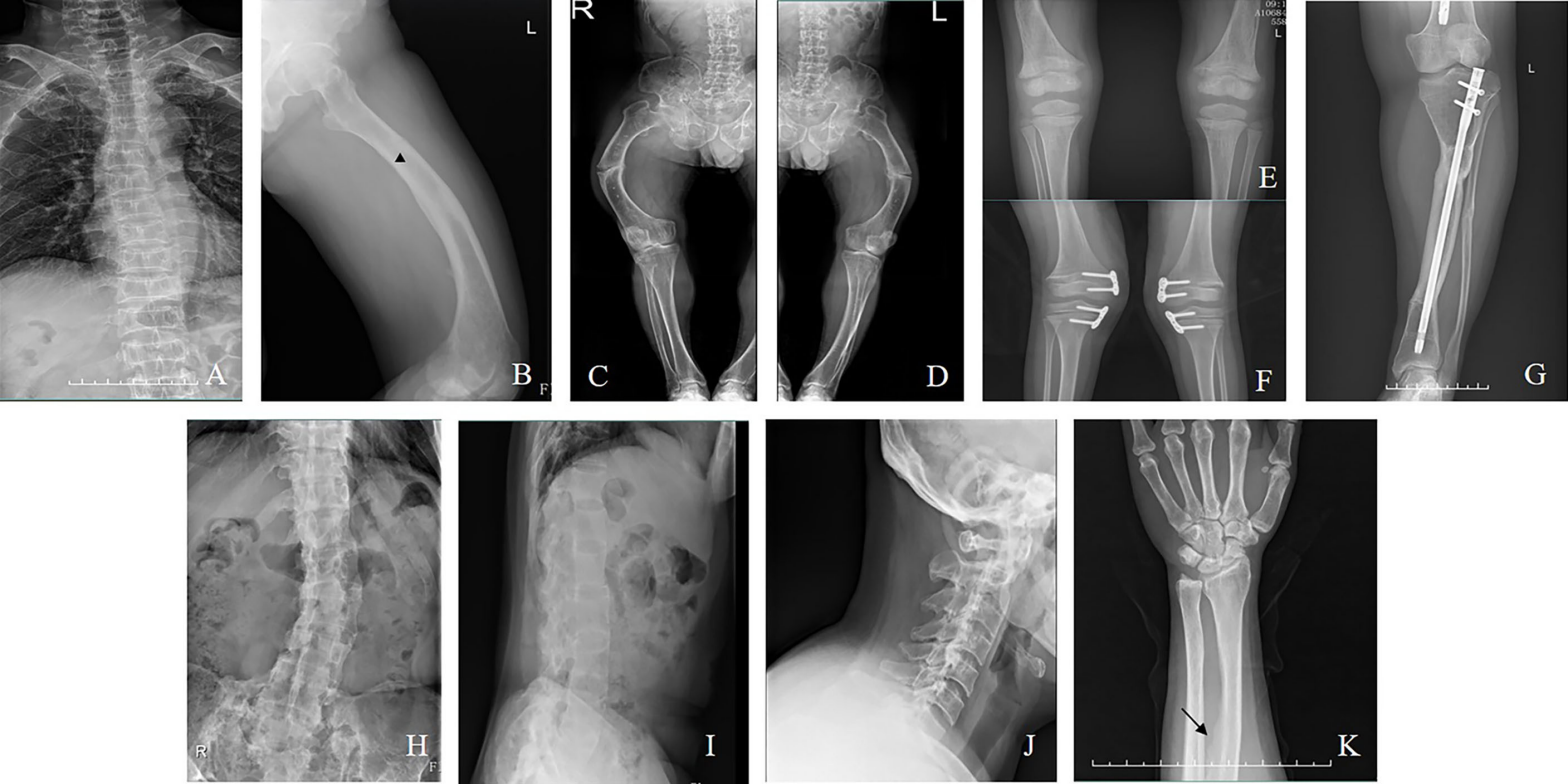
**(A)** Vertebral compression fractures (Family 5/III-8, a man aged 42). **(B)**: Pseudofacture line (Family 4/III-17, a man aged 22). **(C, D)** Fractures in the femur (Family 5/III-8). **(E)** Radiograph of bilateral knee (Family 2/II-4, a boy aged 5). **(F)** Radiograph after hemiepiphysiodesis (Family 9/II-3, a girl aged 7). **(G)** Radiograph after osteotomy (Family 14/III-6, a woman aged 24). **(H–J)** Ossification of the spinal ligaments (Family 14/II-3, a woman aged 50). **(K)** Ossification of the interosseous membrane of the forearm (Family 6/II-4, a woman aged 30).

Manifestations and complications in adult patients are summarized in **Table 2** and **Figure 2**. The majority of patients (12/14) had tooth loss, and half of them reported a history of repeated infections of their teeth, including endodontic infections and periodontitis. Pathological fractures and pseudofractures were detected in four adult patients (including three male patients and one female patient). The common fracture sites were the femur, tibia, and vertebrae. Secondary hyperparathyroidism was detected in seven patients and four patients were diagnosed with ectopic ossification in the spinal ligament, Achilles tendon, and interosseous membrane of the forearm. Before genetic diagnosis, eight patients received conventional treatment with oral neutral phosphorus solution and/or calcitriol. Two of them were diagnosed with nephrocalcinosis *via* renal ultrasound.

### Mutation analysis of the *PHEX* gene

**Figure 3:**
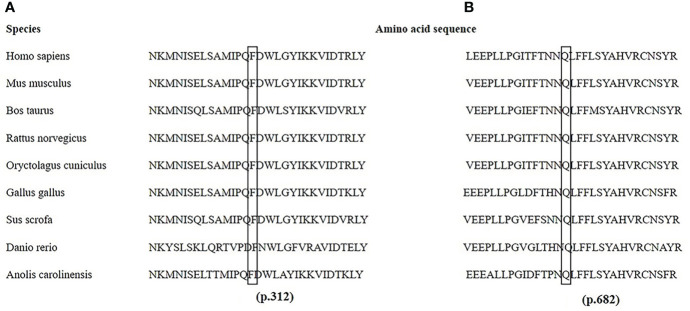
Partial amino acid sequences of the *PHEX* gene from nine species. The amino acids at p.312 in exon 9 **(A)** and p.682 in exon 20 **(B)** are highly conserved in nine different species.

The *PHEX* mutation information is shown in **Supplementary Table 2**. Twenty different mutations were identified, including six missense, three nonsense, two deletion, one insertion, and eight splicing mutations. Two homozygotic mutations (c.350-14_350-1del and c.350_356del) were detected simultaneously in one familial case. Another six novel mutations, c.2045A>T, c.935T>C, c.755_761del, c.1985_1986insTGAC, c.1700+5G>C, and c.1966-1G>T, were identified. Among them, c.2045A>T (p.Gln682Leu) and c.935T>C (p.Phe312Ser) were predicted to be deleterious (PolyPhen-2 score of 1 and 0.99 and PORVEAN score of −6.497 and −4.623, respectively; Mutation Taster score of 0.996 for p.Phe312Ser). Notably, the amino acid residues at p.682 and p.312 were highly evolutionarily conserved across nine different species, as shown in **Figure 3**.

## Discussion

The findings of our study are compatible with previous reports that bowed lower extremities are a common clinical complaint in patients of all ages. Thus, a large number of patients (12/29) underwent at least one surgical limb correction. Bone deformities are usually corrected through one or multiple osteotomies associated with internal or external osteosynthesis (8, 25). Six out of nine adult patients examined in our study underwent this kind of surgery. In this study, the average age of first operation was 11.2 years and the postoperative recurrence rate was 77.8%. Considering the high recurrence rate of the surgery, it may not be appropriate to perform orthopedic surgery before puberty unless serious complications occur. It was suggested that close to the end of puberty with the epiphysis nearly closed would be the best timing for corrective surgeries, which was consistent with previous studies (8, 9, 25, 26). From our study, four adult patients with satisfactory orthopedic surgery underwent their surgeries at the ages of 13, 16, 22, and 30 years, respectively, further supporting our suggestion. Growth-guided surgery, also known as transient hemiepiphysiodesis, is another less invasive technique used to modify deformities in young patients (8). Two children in this study chose this operation. This kind of surgery should be performed with growth potential but is accompanied by risks of recurrence and overcorrection, among others. Considering the strengths and disadvantages of both procedures, the approach of orthopedic surgery depends on the age of the patient and the type of deformity. Moreover, conventional treatment with phosphate and calcitriol before and after surgery was helpful in reducing postoperative recurrence (10, 27). In our study, only two patients received these drugs before surgery, perhaps explaining the high recurrence to some extent.

Regarding clinical features, it should be noted that fractures occurred in familial cases with more males without a history of fall, which could be partially explained by low bone mineral density (BMD, **Table 2**). Ectopic ossification of spinal ligaments and the Achilles tendon was rather prevalent in the adult patients, in addition to secondary hyperparathyroidism and nephrocalcinosis (28). In our study, ossification of the interosseous membrane of the forearm was detected in an adult patient, which was rarely noticed before and could have an adverse impact on the rotation function of the forearm. Therefore, more attention should be paid to adult patients, especially male patients with familial histories, to avoid severe complications.

Furthermore, we identified 20 different mutations in this study. Among them, eight were novel and four were *de novo* mutations. As reported in previous studies, P534L and G579R are mutational hotspots, and they were detected in three cases in our study (11, 29, 30). P534 and G579 are located several amino acids upstream of the active site of the enzyme and conserved zinc-binding domain (including p.580, p.584, and p.642) and are predicted to affect local hydrophobicity and catalytic activity (31, 32). The *PHEX* protein consists of 749 amino acids and we also detected the R747X mutation in a sporadic case, suggesting that even changes in the last three amino acids near the carboxy terminus affect the function of the *PHEX* gene (22).

The eight novel mutations detected here consisted of two nontruncating mutations and six truncating mutations (two deletions, one insertion, two splice acceptor site mutations, and one splice donor site mutation). The two novel missense mutations were p. Gln682Leu in exon 20 and p. Phe312Ser in exon 9, both of which were verified to be pathogenic by bioinformatics tools. Additionally, these sites in p.Gln682 and p.Phe312 were highly evolutionarily conserved among species. Two deletions and one insertion mutation led to premature truncations of p.117, p.252, and p.662, resulting in nonfunctioning *PHEX* products. In addition, three splicing mutations might cause subsequent exons to be skipped, intron retention, or activation of cryptic splice sites, further resulting in a reading frame shift and truncated proteins (33). Notably, homozygous mutations of c.350_356del and c.350-14_350-1del were identified simultaneously in a familial case, emphasizing that all exons and introns should be screened.

Regarding iFGF23 detection, 53.3% of the values were above the upper limit of the reference range (42.2 pg/ml) in our study, which was lower than the percentage reported previously. This discrepancy was partially due to the relatively small sample size and unstable FGF23 protein susceptible to decay in several fragments (22, 29). Neither differences between adult and juvenile patients nor relationships between iFGF23 and clinical phenotypes were detected, in accordance with the findings of previous studies (22, 23, 29). Interestingly, abnormal elevation of iFGF23 (>800 pg/ml) was observed in an adult patient who had been treated with burosumab 2 weeks prior. Previous studies reported that drug therapy could increase the level of iFGF23 to some extent *via* negative feedback (34, 35). However, the mechanism by which iFGF23 increases dramatically in response to burosumab remains to be clarified.

Further studies are needed to further understand the deep-rooted relationship between serum FGF23 and XLH. A case‐control study showed that the concentration of iFGF23 was higher in patients with ossification of the posterior longitudinal ligament than in the control group (36). A latest study revealed higher iFGF23 levels in patients who harbored *PHEX* mutants without zinc-binding sites (31). Meanwhile, potential off-target effects on the cardiovascular, immune, and central nervous systems cannot be ignored given high FGF23 levels *in vivo*, especially in XLH patients with chronic kidney disease (37, 38).

In conclusion, we fully described the clinical and molecular characteristics of 29 patients and discussed the relationships between serum iFGF23 levels and clinical symptoms, common complications, and therapeutic drugs, demonstrating the changes in serum iFGF23 levels after treatment with burosumab in a Chinese patient with XLH. Our study provided an important theoretical basis for expanding the pathogenic gene mutation spectrum of XLH, as well as empirical support for the appropriate timing of orthopedic surgery. Further studies should be performed on a larger sample of patients with XLH in China.

## Data availability statement

The datasets presented in this study can be found in online repositories. The names of the repository/repositories and accession number(s) can be found in the article/**Supplementary Material**.

## Ethics statement

The studies involving human participants were reviewed and approved by the Ethics Committee of Shanghai Jiao Tong University Affiliated Sixth People’s Hospital. Written informed consent to participate in this study was provided by the participants’ legal guardian/next of kin.

## Author contributions

TX drafted the manuscript. TX and XT collected clinical data and blood sample. ZZ, HY, and TX revised the manuscript. ZZ and HY supervised the study. All authors read and approved the final manuscript.

## Funding

This work was supported by the National Key Research and Development Program of China (No. 2018YFA0800801); National Natural Science Foundation of China (NSFC) (Nos.81974126; 81770874); the Clinical Science and Technology Innovation Project of Shanghai Shenkang Hospital Development Center (No. SHDC12018120); Shanghai Key Clinical Center for Metabolic Disease, Shanghai Health Commission Grant (No. 2017ZZ01013).

## Conflict of interest

The authors declare that the research was conducted in the absence of any commercial or financial relationships that could be construed as a potential conflict of interest.

## Publisher’s note

All claims expressed in this article are solely those of the authors and do not necessarily represent those of their affiliated organizations, or those of the publisher, the editors and the reviewers. Any product that may be evaluated in this article, or claim that may be made by its manufacturer, is not guaranteed or endorsed by the publisher.

## References

[1] Laurent MR De Schepper J Trouet D Godefroid N Boros E Heinrichs C. Consensus recommendations for the diagnosis and management of X-linked hypophosphatemia in Belgium. Front Endocrinol (2021) 12:641543. doi: 10.3389/fendo.2021.641543 PMC8018577 33815294

[2] Trombetti A Al-Daghri N Brandi ML Cannata-Andía JB Cavalier E Chandran M. Interdisciplinary management of FGF23-related phosphate wasting syndromes: a consensus statement on the evaluation, diagnosis and care of patients with X-linked hypophosphataemia. Nat Rev Endocrinol (2022) 18:366–84. doi: 10.1038/s41574-022-00662-x 35484227

[3] Bergwitz C Jüppner H. Regulation of phosphate homeostasis by PTH, vitamin d, and FGF23. Annu Rev Med (2010) 61:91–104. doi: 10.1146/annurev.med.051308.111339 PMC4777331 20059333

[4] Gaucher C Walrant-Debray O Nguyen T-M Esterle L Garabédian M Jehan F. PHEX analysis in 118 pedigrees reveals new genetic clues in hypophosphatemic rickets. Hum Genet (2009) 125:401–11. doi: 10.1007/s00439-009-0631-z 19219621

[5] Beck-Nielsen SS Mughal Z Haffner D Nilsson O Levtchenko E Ariceta G. FGF23 and its role in X-linked hypophosphatemia-related morbidity. Orphanet J Rare Dis (2019) 14:58. doi: 10.1186/s13023-019-1014-8 30808384 PMC6390548

[6] Ratsma DMA Zillikens MC van der Eerden BCJ. Upstream regulators of fibroblast growth factor 23. Front In Endocrinol (2021) 12:588096. doi: 10.3389/fendo.2021.588096 PMC7952762 33716961

[7] Gan Y-M Zhang Y-P Ruan D-D Huang J-B Zhu Y-B Lin X-F. Function of PHEX mutations p.Glu145* and p.Trp749Arg in families with X-linked hypophosphatemic rickets by the negative regulation mechanism on FGF23 promoter transcription. Cell Death Dis (2022) 13:518. doi: 10.1038/s41419-022-04969-5 35654784 PMC9163062

[8] Rocco FD Rothenbuhler A Adamsbaum C Bacchetta J Pejin Z Finidori G. Orthopedic and neurosurgical care of X-linked hypophosphatemia. Arch Pediatrie Organe Officiel la Societe Francaise Pediatrie (2021) 28:599–605. doi: 10.1016/j.arcped.2021.09.003 34625380

[9] Horn A Wright J Bockenhauer D Van't Hoff W Eastwood DM. The orthopaedic management of lower limb deformity in hypophosphataemic rickets. J Children's Orthopaedics (2017) 11:298–305. doi: 10.1302/1863-2548.11.170003 PMC5584499 28904636

[10] Novais E Stevens PM. Hypophosphatemic rickets: the role of hemiepiphysiodesis. J Pediatr Orthopedics (2006) 26:238–44. doi: 10.1097/01.bpo.0000218531.66856.b7 16557142

[11] Haffner D Emma F Eastwood DM Duplan MB Bacchetta J Schnabel D. Clinical practice recommendations for the diagnosis and management of X-linked hypophosphataemia. Nat Rev Nephrol (2019) 15:435–55. doi: 10.1038/s41581-019-0152-5 PMC7136170 31068690

[12] Carpenter TO Whyte MP Imel EA Boot AM Högler W Linglart A. Burosumab therapy in children with X-linked hypophosphatemia. N Engl J Med (2018) 378:1987–98. doi: 10.1056/NEJMoa1714641 29791829

[13] Whyte MP Carpenter TO Gottesman GS mao M Skrinar A San martin J. Efficacy and safety of burosumab in children aged 1-4 years with X-linked hypophosphataemia: a multicentre, open-label, phase 2 trial. Lancet Diabetes Endocrinol (2019) 7:189–99. doi: 10.1016/S2213-8587(18)30338-3 30638856

[14] Insogna KL Briot K Imel EA Kamenický P Ruppe MD Portale AA. A randomized, double-blind, placebo-controlled, phase 3 trial evaluating the efficacy of burosumab, an anti-FGF23 antibody, in adults with X-linked hypophosphatemia: Week 24 primary analysis. J Bone Mineral Res (2018) 33:1383–93. doi: 10.1002/jbmr.3475 29947083

[15] Insogna KL Rauch F Kamenický P Ito N Kubota T Nakamura A. Burosumab improved histomorphometric measures of osteomalacia in adults with X-linked hypophosphatemia: A phase 3, single-arm, international trial. J Bone Mineral Res (2019) 34:2183–91. doi: 10.1002/jbmr.3843 PMC6916280 31369697

[16] Imel EA Glorieux FH Whyte MP Munns CF Ward LM Nilsson O. Burosumab versus conventional therapy in children with X-linked hypophosphataemia: A randomised, active-controlled, open-label, phase 3 trial. Lancet (2019) 393:2416–27. doi: 10.1016/S0140-6736(19)30654-3 PMC7179969 31104833

[17] Grund A Sinha MD Haffner D Leifheit-Nestler M. Fibroblast growth factor 23 and left ventricular hypertrophy in chronic kidney disease-a pediatric perspective. Front Pediatr (2021) 9:702719. doi: 10.3389/fped.2021.702719 34422725 PMC8372151

[18] Zheng B Wang C Chen Q Che R Sha Y Zhao F. Functional characterization of PHEX gene variants in children with X-linked hypophosphatemic rickets shows no evidence of genotype-phenotype correlation. J Bone mineral Res (2020) 35:1718–25. doi: 10.1002/jbmr.4035 32329911

[19] Sarafrazi S Daugherty SC Miller N Boada P Carpenter TO Chunn L. Novel PHEX gene locus-specific database: Comprehensive characterization of vast number of variants associated with X-linked hypophosphatemia (XLH). Hum Mutat (2022) 43:143–57. doi: 10.1002/humu.24296 PMC9299612 34806794

[20] Li H Ji C-Y Zong X-N Zhang Y-Q. [Height and weight standardized growth charts for Chinese children and adolescents aged 0 to 18 years]. Zhonghua er ke za zhi = Chin J Pediatr (2009) 47:487–92. doi: 10.3760/cma.j.issn.0578-1310.2009.07.003 19951507

[21] Thacher TD Pettifor JM Tebben PJ Creo AL Skrinar A Mao M. Rickets severity predicts clinical outcomes in children with X-linked hypophosphatemia: Utility of the radiographic rickets severity score. Bone (2019) 122:76–81. doi: 10.1016/j.bone.2019.02.010 30772600

[22] Zhang C Zhao Z Sun Y Xu L Jiajue R Cui L. Clinical and genetic analysis in a large Chinese cohort of patients with X-linked hypophosphatemia. Bone (2019) 121:212–20. doi: 10.1016/j.bone.2019.01.021 30682568

[23] Li S-S Gu J-M Yu W-J He J-W Fu W-Z Zhang Z-L. Seven novel and six *de novo* PHEX gene mutations in patients with hypophosphatemic rickets. Int J Mol Med (2016) 38:1703–14. doi: 10.3892/ijmm.2016.2796 PMC5117772 27840894

[24] Hu W-W Zhang Z He J-W Fu W-Z Wang C Zhang H. Establishing reference intervals for bone turnover markers in the healthy shanghai population and the relationship with bone mineral density in postmenopausal women. Int J Endocrinol (2013) 2013:513925. doi: 10.1155/2013/513925 23533403 PMC3600195

[25] Gizard A Rothenbuhler A Pejin Z Finidori G Glorion C De Billy B. Outcomes of orthopedic surgery in a cohort of 49 patients with X-linked hypophosphatemic rickets (XLHR). Endocrine connections (2017) 6:566–73. doi: 10.1530/EC-17-0154 PMC5633063 28954742

[26] Song H-R Soma Raju VVJ Kumar S Lee S-H SUH S-W KIM J-R. Deformity correction by external fixation and/or intramedullary nailing in hypophosphatemic rickets. Acta Orthopaedica (2006) 77:307–14. doi: 10.1080/17453670610046073 16752295

[27] Petje G Meizer R Radler C Aigner N Grill F. Deformity correction in children with hereditary hypophosphatemic rickets. Clin Orthopaedics Related Res (2008) 466:3078–85. doi: 10.1007/s11999-008-0547-2 PMC2628230 18841431

[28] Kato H Koga M Kinoshita Y Taniguchi Y Kobayashi H Fukumoto S. Incidence of complications in 25 adult patients with X-linked hypophosphatemia. J Clin Endocrinol Metab (2021) 106:e3682–92. doi: 10.1210/clinem/dgab282 33912912

[29] Lin X Li S Zhang Z Yue H. Clinical and genetic characteristics of 153 Chinese patients with X-linked hypophosphatemia. Front Cell Dev Biol (2021) 9:617738. doi: 10.3389/fcell.2021.617738 34141703 PMC8204109

[30] Sabbagh Y Boileau G Desgroseillers L Tenenhouse HS. Disease-causing missense mutations in the PHEX gene interfere with membrane targeting of the recombinant protein. Hum Mol Genet (2001) 10:1539–46. doi: 10.1093/hmg/10.15.1539 11468271

[31] Ishihara Y Ohata Y Takeyari S Kitaoka T Fujiwara M Nakano Y. Genotype-phenotype analysis, and assessment of the importance of the zinc-binding site in PHEX in Japanese patients with X-linked hypophosphatemic rickets using 3D structure modeling. Bone (2021) 153:116135. doi: 10.1016/j.bone.2021.116135 34333162

[32] Durmaz E Zou M Al-Rijjal RA Baitei EY Hammami S Bircan I. Novel and *de novo* PHEX mutations in patients with hypophosphatemic rickets. Bone (2013) 52:286–91. doi: 10.1016/j.bone.2012.10.012 23079138

[33] Binessa HA Zou M Al-Enezi AF Alomrani B Al-Faham MSA Al-Rijjal RA. Functional analysis of 22 splice-site mutations in the PHEX, the causative gene in X-linked dominant hypophosphatemic rickets. Bone (2019) 125:186–93. doi: 10.1016/j.bone.2019.05.017 31102713

[34] Imel EA Dimeglio LA Hui SL Carpenter TO Econs MJ. Treatment of X-linked hypophosphatemia with calcitriol and phosphate increases circulating fibroblast growth factor 23 concentrations. J Clin Endocrinol Metab (2010) 95:1846–50. doi: 10.1210/jc.2009-1671 PMC2853995 20157195

[35] Piketty M-L Brabant S Souberbielle J-C Maruani G Audrain C Rothenbuhler A. FGF23 measurement in burosumab-treated patients: an emerging treatment may induce a new analytical interference. Clin Chem Lab Med (2020) 58:e267–9. doi: 10.1515/cclm-2020-0460 32653872

[36] Kawaguchi Y Kitajima I Nakano M Yasuda T Seki S Suzuki K. Increase of the serum FGF-23 in ossification of the posterior longitudinal ligament. Global Spine J (2019) 9:492–8. doi: 10.1177/2192568218801015 PMC6686384 31431871

[37] Pastor-Arroyo E-M Gehring N Krudewig C Costantino S Bettoni C Knöpfel T. The elevation of circulating fibroblast growth factor 23 without kidney disease does not increase cardiovascular disease risk. Kidney Int (2018) 94:49–59. doi: 10.1016/j.kint.2018.02.017 29735309

[38] Vervloet M. Renal and extrarenal effects of fibroblast growth factor 23. Nat Rev Nephrol (2019) 15:109–20. doi: 10.1038/s41581-018-0087-2 30514976

