# Supplementary material for: Clinical and genetic characteristics of 29 Chinese patients with X-linked hypophosphatemia
Source: Front Endocrinol (Lausanne). 2022 Aug 19;13:956646. doi: 10.3389/fendo.2022.956646 (PMC9437435; doi:10.3389/fendo.2022.956646)
Supplement: Supplementary file 1 [file DataSheet_1.pdf]

Supplementary Table 1

Family No.	Patient number/Gender	Age(years)	Age of onset(years)	Height SDS	Weight (kg)	BMI（kg/m^2）	β-CTX (ng/ml)	OC (ng/ml)	PTH(pg/ml)	25OHD (ng/ml)	Ca (mmol/L)	P (mmol/L)	ALP (U/L)	Cr (μmol/L)	iFGF23(pg/ml)
1	II-5/M	27.0	6.0	-5.84	41.0	20.92	645.0	25.93	113.6	11.21	2.48	0.48	172	49.0	13.8
1	III-7/F	9.0	-	-	-	-	2466.0	137.60	215.2	13.55	2.36	0.83	597	43.0	8.5
2	II-4/M	5.0	1.0	-2.00	18.6	17.53	3450.0	254.80	866.0	29.87	2.30	1.16	868	26.9	282.3
2	I-2/F	44.0	4.0	-5.66	44.9	25.77	371.2	18.25	64.38	13.33	2.19	0.53	136	34.2	171.7
3	III-12/F	0.6	-	+0.09	6.5	14.48	1962.0	43.78	14.09	65.46	2.73	1.00	425	31.4	
3	III-11/M	1.0	1.0	-2.83	9.0	18.90	2240.0	147.00	52.57	57.80	2.51	0.84	547	29.6	
4	III-17/M	22.0	2.0	-6.73	60.0	32.92	2134.0	91.47	103.60	13.55	2.28	0.45	480	39.8	
5	III-8/M	42.0	1.0	-5.84	46.0	23.47	846.0	35.79	94.52	12.72	2.32	0.45	210	67.6	
6	II-5/M	24.0	1.0	-6.73	52.0	28.53	1988.0	58.40	83.37	8.08	2.23	0.51	268	43.1	
6	II-4/F	30.0	3.0	-3.09	45.5	21.64	548.9	19.82	44.24	20.88	2.17	0.55	104	49.3	
6	III-6/F	6.0	1.0	-1.50	23.5	19.42	2940.0	101.30	82.91	30.13	2.27	0.76	652	29.8	22.9
7	I-2/F	34.0	1.0	-2.10	48.0	21.33	-	-	-	-	-	-	-	-	
7	II-3/M	0.4	-	-0.17	8.5	18.38	-	-	-	60.80	2.48	1.00	786	-	
8	II-3/M	33.0	2.0	-1.38	67.0	24.61	-	-	-	-	-	-	-	-	55.7
8	III-5/F	3.0	1.0	-0.16	15.5	17.17	1741.0	81.95	35.07	46.11	2.39	0.75	505	29.3	136.3
9	II-3/F	7.0	2.0	-1.77	21.0	16.16	1931.0	67.43	71.83	33.01	2.28	1.03	874	37.4	24.0
10	III-9/M	12.0	2.0	-2.57	33.1	18.49	3356.0	281.7	169.4	24.73	2.48	0.67	716	30.1	146.1
10	II-5/F	39.0	1.0	-4.67	47.0	25.04	357.2	21.20	49.39	21.77	2.29	0.71	119	38.7	
11	II-3/M	21.0	2.0	-3.52	62.0	26.49	1361.0	47.56	71.61	24.84	2.34	0.61	75	53.3	40.4
12	II-3/F	27.0	1.0	-2.99	61.9	29.24	721.7	25.01	46.50	21.15	2.36	0.68	124	45.6	58.6
13	II-3/F	3.01	1.0	-0.99	14.5	17.13	1814.0	81.94	42.35	49.71	2.42	0.85	500	28.8	

14	III-6/F	24.0	1.0	-3.09	52.6	25.02	225.7	20.45	51.87	30.11	2.28	0.76	58	40.2	
14	II-3/F	50.0	2.0	-6.61	58.7	36.28	-	-	77.50	15.20	2.21	0.70	102	40.0	
15	II-3/M	5.0	3.0	+0.17	21.0	16.74	-	-	59.60	26.18	2.30	-	338	23.0	
16	II-4/F	9.0	3.0	-0.84	25.0	14.91	345.0	-	64.00	40.50	2.28	0.89	497	43.9	151.5
17	II-3/M	11.0	5.0	-1.14	50.0	26.25	2064.0	125.90	99.33	23.37	2.30	0.97	521	26.1	63.1
18	II-4/M	16.0	5.0	-3.72	80.0	35.56	1883.0	63.10	28.17	13.52	2.59	0.59	302	34.6	26.7
19	II-3/F	9.0	1.0	-1.45	26.0	16.35	-	-	111.30	-	2.36	0.96	382	-	
20	II-3/F	23.0	5.0	-3.68	36.0	17.85	698.0	18.10	78.02	9.85	2.22	0.68	124	51.8	37.4

Supplementary Table 2

Family No.	Inheritance	Mutation sites	PHEX mutation
1	Familial	Exon 21	Arg702X(c.2104C>T)
2	Familial	Intron 16	c.1700+5G>C
3	Familial	Intron 14	c.1586+3G>T
4	Familial	Exon 15	Ile548Thr(c.1643T>C)
5	Familial	Exon 15	Pro534Leu(c.1601C>T)
6	Familial	Intron 3	c.350-14_350-1del
		Exon 4	Glu117GlyfsX25(c.350_356del)
7	Familial	Intron 17	c.1768+1G>A
8	Familial	Exon 6	Leu222Pro(c.665T>C)
9	Sporadic	Exon 15	Pro534Leu(c.1601C>T)
10	Familial	Exon 7	Phe252TrpfsX7(c.755_761del)
11	Sporadic	Exon 20	Gln682Leu(c.2045A>T)
12	Sporadic	Intron 19	c.1966-1G>T
13	Sporadic	Exon 9	Phe312Ser(c.935T > C)
14	Familial	Intron 11	c.1302+1G > A
15	Sporadic	Exon 20	Asn662fs(c.1985_1986insTGAC)
16	Sporadic	Exon 17	Gly579Arg(c.1735G > A)
17	Sporadic	Exon 22	Arg747*(c.2239C > T)
18	Familial	Exon 16	Arg567X(c.1699C>T)
19	Familial	Intron 2	c.187+1_4delGTAA
20	Sporadic	Intron 15	c.1645+1G>A
